# A Literature Review of Gene Function Prediction by Modeling Gene Ontology

**DOI:** 10.3389/fgene.2020.00400

**Published:** 2020-04-24

**Authors:** Yingwen Zhao, Jun Wang, Jian Chen, Xiangliang Zhang, Maozu Guo, Guoxian Yu

**Affiliations:** ^1^College of Computer and Information Science, Southwest University, Chongqing, China; ^2^State Key Laboratory of Agrobiotechnology and National Maize Improvement Center, China Agricultural University, Beijing, China; ^3^CBRC, King Abdullah University of Science and Technology, Thuwal, Saudi Arabia; ^4^School of Electrical and Information Engineering, Beijing University of Civil Engineering and Architecture, Beijing, China

**Keywords:** gene ontology, gene function prediction, functional genomics, directed acyclic graph, inter-relationships, semantic similarity

## Abstract

Annotating the functional properties of gene products, i.e., RNAs and proteins, is a fundamental task in biology. The Gene Ontology database (GO) was developed to systematically describe the functional properties of gene products across species, and to facilitate the computational prediction of gene function. As GO is routinely updated, it serves as the gold standard and main knowledge source in functional genomics. Many gene function prediction methods making use of GO have been proposed. But no literature review has summarized these methods and the possibilities for future efforts from the perspective of GO. To bridge this gap, we review the existing methods with an emphasis on recent solutions. First, we introduce the conventions of GO and the widely adopted evaluation metrics for gene function prediction. Next, we summarize current methods of gene function prediction that apply GO in different ways, such as using hierarchical or flat inter-relationships between GO terms, compressing massive GO terms and quantifying semantic similarities. Although many efforts have improved performance by harnessing GO, we conclude that there remain many largely overlooked but important topics for future research.

## 1. Introduction

Functional annotations of gene products, i.e., proteins and RNAs, can promote the progress of drug development (Barabási et al., 2011; Xuan et al., 2019), disease analysis (Kissa et al., 2015; Zeng et al., 2015; Zhang et al., 2019), gene set enrichment analysis (Zheng and Wang, 2008; Mi et al., 2013), and many other domains (Radivojac et al., 2013; Jiang et al., 2016; Shehu et al., 2016; Zhou et al., 2019). Advances in bio-technology make it possible to perform high-throughput experiments, which yield diverse functional information about gene products, at decreasing costs. The key task has shifted from collecting such data to analyzing the data with a unified functional description scheme. To address this problem, some paradigms (Ashburner et al., 2000; Ruepp et al., 2004; Dessimoz and Škunca, 2017) aim to describe the functional properties of gene products in a formal and species neutral way, as well as to assist computational gene function prediction. Among these paradigms, Gene Ontology (GO) (Ashburner et al., 2000) and MIPS Functional Catalog (FunCat) (Ruepp et al., 2004) are the most often used. Compared with FunCat, GO is more comprehensive, is continuously updated, has more affiliated functional annotations, and is more widely used. Therefore, we focus on function prediction methods using GO.

**Figure 1:**
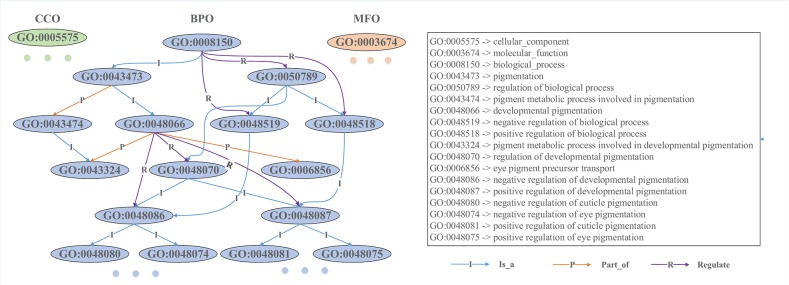
Snapshot of a directed acyclic graph from Gene Ontology. Each ontological term is represented by an alphanumeric identifier, and its biological function is described by controlled words. These GO terms are hierarchically connected with different types of directed edges. The level of a GO term in the DAG is determined by its furthest distance to the root GO term (“GO:0008150” in BPO, “GO:0005575” in CCO, and “GO:0003674” in MFO). For example, “GO:0048087” is a direct child and also a grandson of “GO:0048066,” and its furthest distance to the root term is 5, while “GO:0006856” is another direct child of “GO:0048066” and its furthest distance to the root is 4, so “GO:0006856” is plotted at a higher level than “GO:0048087”.

GO is composed of three ontologies: molecular functional ontology (MFO), biological process ontology (BPO), and cellular component ontology (CCO) (Ashburner et al., 2000). MFO describes the elemental activities of a gene product at the molecular level (i.e., binding and catalysis); BPO captures the beginning and end, pertinent to the functioning of integrated living units: cells, tissues, organs, and organisms; CCO describes the parts of cells and their extracellular environments. Each ontology consists of a set of ontological terms (GO terms), which are organized in a hierarchy, or directed acyclic graph (DAG), as shown in Figure 1. This DAG can be generated from the ontology file with moderate scripts (i.e., Matlab, R, and Python). In the Supplementary Material, we provide some exemplar codes for generating an association matrix from GO and to visualize the Ontology. Each GO term is defined by a unique alphanumeric identifier and can be viewed as a vertex of the graph, and the function is described using controlled words. The edge encodes the relationships (*is a, part of*, and *regulate*) between GO terms. For example, “GO:0043473” represents the pigmentation, and “GO:0048066” describes the developmental pigmentation; the two terms are connected by a line with “I,” which means that the developmental pigmentation *is a* subtype of pigmentation.

GO annotation is another component of GO, and it stores the *currently* known functional knowledge of gene products. Each positive annotation relates a gene with a GO term, and indicates the gene product carries out the function described by this term. Similarly, each negative annotation indicates the gene product does not perform the function described by this term. The GO consortium (Ashburner et al., 2000) independently or collaboratively annotate genes with GO terms from model organisms (or species) of wide interest among biologists, such as *Homo sapiens, Mus musculus, Arabidopsis thaliana*, and so on. However, our current knowledge about the functional taxonomy of gene products is still immature. Therefore, both the GO hierarchy and annotations are regularly updated with new knowledge and archived for reference. The collected GO annotations are still quite incomplete, imbalanced, and rather shallow (Rhee et al., 2008; Thomas et al., 2012; Dessimoz and Škunca, 2017). For example, different species have different distributions of GO annotations; *zebrafish* is heavily studied in terms of developmental biology and embryogenesis, while *rat* is the standard model for toxicology (Dessimoz and Škunca, 2017). The portion of negative annotations is much smaller than positive ones, because a negative result may be due to inadequate experimental conditions and is often deemed as less useful and publishable than a positive annotation. By December 2019, GO included more than 45,000 terms, and each gene was only annotated with several or dozens of these terms. Therefore, it is rather difficult to accurately infer the associations between the genes and the many GO terms.

Each GO term can be modeled as a semantic label and, thus, the gene function prediction task can be treated as a classification problem to determine whether the label is positive for the gene or not. Early gene function prediction solutions simply utilized this annotation information (Schwikowski et al., 2000; Hvidsten et al., 2001; Raychaudhuri et al., 2002; Schug et al., 2002; Troyanskaya et al., 2003; Karaoz et al., 2004), and converted the problem into a plain binary (or multi-class) classification task (Hua and Sun, 2001; Lanckriet et al., 2003; Leslie et al., 2004). Such methods ignored the correlations between the GO terms and the imbalanced characteristics of terms; therefore, their accuracy was low. Since a gene is often simultaneously annotated with a set of structurally organized GO terms, some researchers model gene function prediction as a multi-label or structural output prediction task (Barutcuoglu et al., 2006; Obozinski et al., 2008; Zhang and Zhou, 2014; Kahanda and Ben-Hur, 2017). Others attempted to use the inter-relationships among GO terms, and introduced a variety of solutions based on multi-label learning. These generally obtained an improved accuracy (Mostafavi et al., 2008; Mostafavi and Morris, 2010; Yu et al., 2012a, 2015a).

**Figure 2:**
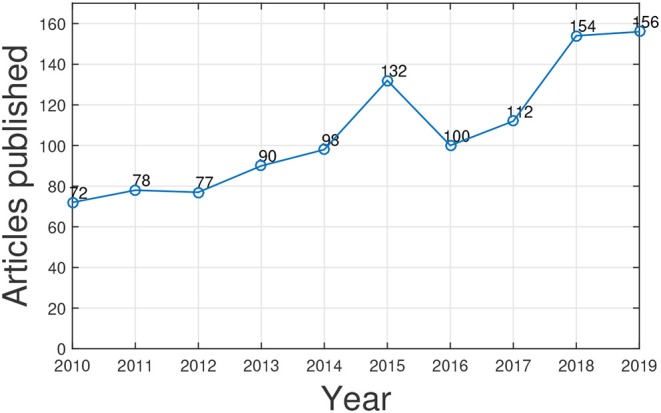
The number of published papers related to GO-based gene function prediction over 10 years.

We utilized Web of Science^1^ to search articles related to gene function prediction using GO published in the past 10 years through a keyword search: “gene ontology and gene function prediction.” The statistic counts are shown in Figure 2. We can see that research interest in this topic is increasing. As the need of human knowledge (i.e., GO and its annotations) for artificial intelligence in biology increases, we believe the study of GO for gene function prediction and for other biomedical data mining tasks will be fast growing. Several excellent surveys provide a comprehensive literature summation of the progress in gene function prediction (a.k.a. protein function prediction) and the studies of GO from different perspectives (Pandey et al., 2006; Tiwari and Srivastava, 2014; Valentini, 2014; Mazandu et al., 2016; Shehu et al., 2016; Dessimoz and Škunca, 2017). However, to the best of our knowledge, none of them focus on harnessing GO for gene function prediction.

**Figure 3:**
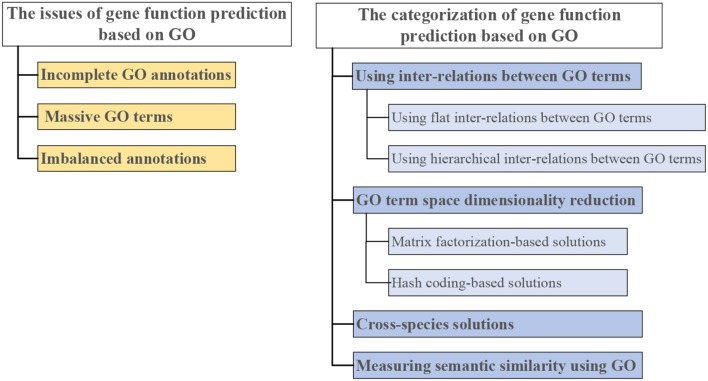
Three issues in gene function prediction **(left)**, and categorization of existing computational solutions based on GO **(right)**.

Therefore, we give a comprehensive review of GO-based gene function prediction methods ( categorized in Figure 3). The three main issues in gene function prediction are summarized on the left side of Figure 3. Categories of computational methods that combat one or two of these issues are on the right side of Figure 3. Each of these methods is detailed in the following sections.

The rest of this review is organized as follows. We introduce the workflow of gene function prediction, conventions in GO and typical evaluation metrics in section 2. In section 3, we categorize the existing GO-based gene function prediction methods. In section 4, we summarize remaining issues, as well as some interesting but less explored topics in gene function prediction. Section 5 concludes the survey.

## 2. Related Knowledge

Gene function prediction methods mainly utilize the structure of GO and biological features (including nucleotide/amino acids sequences, gene expression, and interaction data, etc.) of genes. Therefore, we first review the basic workflow of gene function prediction, introduce the *True Path Rule*, and evidence codes from GO, and then present the widely-used evaluation metrics for gene function prediction.

### 2.1. The Workflow of Gene Function Prediction

The GO file and annotation files are publicly accessible at http://geneontology.org/. They are regularly updated and archived. GO can be represented by a DAG (**G**∈ℝ^*m* × *m*^ for *m* terms). The GO annotations are usually encoded by a gene-term association matrix (**Y**∈ℝ^*n* × *m*^ for *n* genes with respect to *m* GO terms). If gene *i* is annotated with *t* or *t*'s descendants, then **Y**(*i, t*) = 1; if this gene is not annotated with *t* or its ancestor, then **Y**(*i, t*) = −1; otherwise, **Y**(*i, t*) = 0. We want to remark that **Y**(*i, t*) = 0 simply indicates that till now there is no evidence that this gene does or does not carry out the function related to term *t*. This specification is based on the incompleteness and open-world assumption of GO annotations (Schnoes et al., 2013; Dessimoz and Škunca, 2017). If **X**∈ℝ^*n* × *d*^ stores the numeric features of these genes, then the function prediction task can be seen as a classification task that makes use of **Y** and input pattern **X** to train a model, which can predict the association probabilities between these (or new) genes and GO terms.

**Figure 4:**
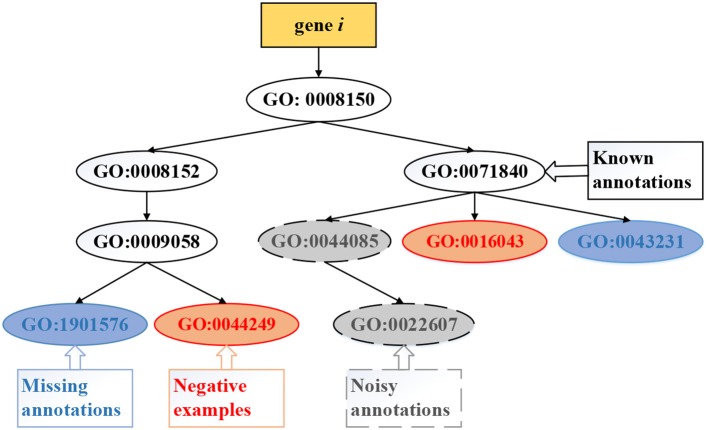
Exemplar tasks of gene function prediction, which include predicting *missing, negative*, and *noisy* annotations.

Existing methods of computational gene function prediction generally focus on the three tasks ( illustrated in Figure 4): (i) predicting *missing* (new) annotations, which updates some entries in **Y** with value 0 into 1 to identify new functional annotations of genes; (ii) identifying *noisy* annotations, which updates some entries in **Y** with value 1 into −1 to remove these false positive annotations; (iii) predicting *negative* examples, which updates some entries in **Y** with value 0 into −1 to state that the gene clearly does not carry out this function. The first task has been extensively studied, while the latter two tasks are attracting research interest.

The evaluation protocol for gene function prediction is generally performed one of two ways. One way is called *history to recent*, which takes advantage of previously archived GO annotations to train a model and evaluate the model's predictions by referring to more recent GO annotations. The second way is called *dataset partition* (or cross-validation), which divides the archived GO annotations into two (or three) sets, the first one (or two) sets for training (or tuning) the predictor, and the remaining set for testing the predictor. There are three main differences between the two ways. First, from the view of selecting training and testing sets, the *history to recent* evaluation is affected by the time span, since GO annotations are regularly updated. A time span of one or 2 years is often adopted. The *dataset partition* evaluation is influenced by the proportion of training and testing sets; a higher proportion of training sets generally gives better results. Second, from the prediction results, the *history to recent* way evaluates the fixed, recent annotations and, thus, it does not have a variance. In contrast, the *dataset partition* evaluation has to repeat multiple, independent runs to avoid the impact of random partition, and the average results and variances are both influenced. The results obtained in the *history to recent* evaluation are generally better than those obtained by the *dataset partition* evaluation. That is because *history to recent* evaluation uses all the genes and annotations for training, while *dataset partition* only uses genes in the training set and excludes genes in the testing set. Third, from the application view, the *history to recent* evaluation is deemed as more realistic and is more popular. Since GO annotations are regularly updated, the *history to recent* can reflect the potential of the model with up-to-data annotations. In contrast, the *dataset partition* may suffer from a circular prediction caused by the complex inter-connections between the partitioned training and testing sets.

### 2.2. Conventions in GO

#### 2.2.1. True Path Rule

The *True Path Rule* is one of the most important rules in GO (Blake, 2013), and should be respected in gene function prediction. If a gene is annotated with GO term *t*, then this gene is also annotated with *t*'s ancestor terms. Conversely, if this gene does not have the function described by *t*, then it should not be annotated with *t*'s descendant terms other. From this rule, we have

(1) $$\begin{array}{r} p\left(t|par\left(t\right)\right)\geq p\left(t|gpar\left(t\right)\right) \end{array}$$

(2) $$\begin{array}{r} p\left(t|gpar\left(t\right)\right)\geq p\left(t|uncle\left(t\right)\right) \end{array}$$

where *par*(*t*) denotes the parent term of term *t*, *gpar*(*t*) is the grandparent term of *t*, and *uncle*(*t*) is the uncle (parent's sibling) term of *t*. *p*(*t*|*par*(*t*)) is the conditional probability that a gene is annotated with *t* given this gene is already annotated with *par*(*t*). These equations imply that if a gene is annotated with GO terms *par*(*t*) [or *uncle*(*t*)], then this gene is also annotated with *gpar*(*t*) (if any), but not vice versa.

Given the structural relationships between terms, gene function prediction can be viewed as a structure output or multi-label learning problem (Barutcuoglu et al., 2006; Obozinski et al., 2008; Yu et al., 2012a; Zhang and Zhou, 2014; Kahanda and Ben-Hur, 2017; Kulmanov et al., 2017). The structure or multi-label predictions are consistent if they obey the *True Path Rule* or satisfy Equations (1, 2). According to this rule, a positive prediction for a term but a negative prediction for its ancestor terms with respect to the same gene are inconsistent predictions. In other words, a positive prediction for a term implies positive predictions for all the ancestors, and a negative prediction implies negative associations for all the descendant terms.

#### 2.2.2. Evidence Code

Each GO annotation is tagged with one or more evidence codes, which state the type of evidence (or source) from which the annotation is collected. GO adopts 21 evidence codes and groups them into four categories: (i) *Experimental*: EXP (Inferred from Experiment), IDA (Inferred from Direct Assay), IPI (Inferred from Physical Interaction), IMP (Inferred from Mutant Phenotype), IGI (Inferred from Genetic Interaction), and IEP (Inferred from Expression Pattern); (ii) *Computational*: ISS (Inferred from Sequence or structural Similarity), ISO (Inferred from Sequence Orthology), ISA (Inferred from Sequence Alignment), ISM (Inferred from Sequence Model), IGC (Inferred from Genomic Context), IBA (Inferred from Biological aspect of Ancestor), IBD (Inferred from Biological aspect of Descendant), IKR (Inferred from Key Residues), IRD (Inferred from Rapid Divergence), RCA (Inferred from Reviewed Computational Analysis), and IEA (Inferred from Electronic Annotation); (iii) *Author*: TAS (Traceable Author Statement) and NAS (Non-traceable Author Statement); (iv) *Curatorial*: IC (Inferred by Curator) and ND (No biological Data Available) (Consortium et al., 2017). The specific meanings of these evidence codes can be found at http://www.geneontology.org/page/guide-go-evidence-codes.

Except IEA, all other evidence codes are curated by curators. Several studies investigate the quality of GO annotations from the perspective of evidence codes. Thomas et al. (2007) proposed to apply evidence codes as indicator for the reliability of annotations, and found that the annotations achieved by experimental and author statement are more reliable than others. Clark and Radivojac (2011) investigated the quality of NAS and IEA annotations, and found IEA annotations were much more reliable than NAS ones in MFO branch. Gross et al. (2009) considered evolutionary changes to evaluate stability and quality of different evidence codes. Buza (2008) estimated the annotation quality with respect to terms in BPO via a rank of evidence codes. Jones et al. (2007) found that a high false positive rate is obtained when leveraging ISS annotations and sequence data as the basis for prediction. Yu et al. (2017c) adopted evidence codes to weight the annotations and to identify the noisy annotations.

### 2.3. Evaluation Metrics

Multiple evaluation metrics can be adopted to quantify the results of gene function prediction. Given the complexity of gene function prediction, these metrics aim to evaluate the performance from different aspects (Radivojac et al., 2013; Jiang et al., 2016). For recent gene function prediction, *AUC, Fmax*, and *Smin* are recommended by CAFA (Critical Assessment of protein Function Annotation algorithms) (Radivojac et al., 2013; Jiang et al., 2016; Zhou et al., 2019). *AUC* defines different thresholds to plot the receiver-operating characteristics curve of each GO term, and then calculates the average-area value of these terms.

*Fmax* is the overall maximum harmonic mean of precision and recall across all possible thresholds on the predicted gene-term association matrix (Jiang et al., 2016). The formal definition of *Fmax* is

(3) $$\begin{array}{r} Fmax=\underset{\theta }{\max}\frac{2pre\left(\theta \right)rec\left(\theta \right)}{pre\left(\theta \right)+rec\left(\theta \right)} \end{array}$$

(4) $$\begin{array}{r} pre\left(\theta \right)=\frac{1}{m\left(\theta \right)}\overset{m\left(\theta \right)}{\underset{i=1}{\sum }}\frac{TP_{i}}{TP_{i}+FP_{i}} \end{array}$$

(5) $$\begin{array}{r} rec\left(\theta \right)=\frac{1}{n}\overset{n}{\underset{i=1}{\sum }}\frac{TP_{i}}{TP_{i}+FN_{i}} \end{array}$$

where *m*(θ) is the number of genes, which have at least one predicted score ≥ θ. *TP*_*i*_ counts the number of true positive predictions, *FP*_*i*_ is the number of false positive predictions and *FN*_*i*_ counts the number of false negative predictions for gene *i*.

*Smin* utilizes information theoretic analogs based on the GO hierarchy to evaluate the minimum semantic distance between the predictions and ground-truths across all possible thresholds (Jiang et al., 2014). The formal definition of *Smin* is

(6) $$\begin{array}{r} Smin=\underset{\theta }{\min}\sqrt{ru{\left(\theta \right)}^{2}+mi{\left(\theta \right)}^{2}} \end{array}$$

(7) $$\begin{array}{r} ru\left(\theta \right)=\frac{1}{n}\overset{n}{\underset{i=1}{\sum }}\underset{t}{\sum }IC\left(t\right)l\left(t\notin p_{i}\left(\theta \right)\wedge t\in T_{i}\right) \end{array}$$

(8) $$\begin{array}{r} mi\left(\theta \right)=\frac{1}{n}\overset{n}{\underset{i=1}{\sum }}\underset{t}{\sum }IC\left(t\right)l\left(t\in p_{i}\left(\theta \right)\wedge t\notin T_{i}\right) \end{array}$$

where *IC*(*t*) is the information content of the term *t*, which estimates a term's specificity by its frequency of annotation to genes (Lin, 1998). *p*_*i*_(θ) denotes the set of terms with predicted scores ≥ θ for gene *i*, and *T*_*i*_ denotes the set of terms annotated to that gene. In addition, the area under the precision-recall curve (AUPRC) is also widely used as an evaluation metric. Unlike AUC, it accounts for the imbalance in the GO terms and is also more discriminant than AUC (Guan et al., 2008; Peña-Castillo et al., 2008).

Gene function prediction can be viewed as a multi-label classification problem (Yu et al., 2012a; Zhang et al., 2012). Evaluation metrics for multi-label learning are also used to quantify the performance of gene function prediction, such as *MicroAvgF1, MacroAvgF1, RankingLoss, Coverage*, and *AvgPrecision*. *MicroAvg-F1* calculates the F1 measure from the predictions of different GO terms as a whole; it is more affected by the performance of terms that have more relevant genes. *MacroAvgF1* averages the F1 scores of different GO terms, and is more affected by the performance of sparse GO terms with fewer relevant genes. *RankingLoss* evaluates the average fraction of GO-term pairs that are incorrectly ranked. *Coverage* examines the search steps to cover all relevant annotations from a predicted gene-term association matrix. *AvgPrecision* evaluates the average fraction of GO terms ranked above a particular GO term. The formal definitions of these multi-label evaluation metrics can be found elsewhere (Zhang and Zhou, 2014; Gibaja and Ventura, 2015). Here, we want to highlight that these metrics quantify the results of gene function prediction from different perspectives. Any single prediction model generally cannot consistently outperform all others across each of these metrics.

## 3. Categorization of Existing Solutions

It is difficult to give a pure categorization of GO-based gene function prediction solutions since there are always overlaps. In this paper, we classify the existing solutions according to whether hierarchical inter-relations are used between the GO terms, and whether the massive GO terms are compressed.

### 3.1. Gene Function Prediction Using Inter-Relations Between GO Terms

GO uses a DAG to hierarchically organize the GO terms. This DAG encodes domain knowledge of biology. Evidence suggests that using the inter-relations between GO terms can boost the performance of gene function prediction (Tao et al., 2007; Pandey et al., 2009; Done et al., 2010). The inter-relations between GO terms can be measured from different viewpoints (Teng et al., 2013; Peng et al., 2018), and can be roughly grouped into two categories, *flat* and *hierarchical*. The flat inter-relations simply consider the occurrence of two GO terms annotated to the same genes, without explicitly using the hierarchical structure between the terms. The hierarchical inter-relations additionally account for the ontology structure. Based on the target tasks, we further divide those two methods into three subtypes based on whether they predict missing, noisy or negative annotations of genes, as listed in Table 1.

**Table 1:** Categories of solutions that use different inter-relations between GO terms.

	**Solutions**	**Inter-relations**	**Basic techniques**
Predicting *missing* annotations	ProWL (Yu et al., 2012b)	Flat	Weak label learning
	ProDM (Yu et al., 2013a)	Flat	Weak label learning
	ProHG (Liu et al., 2016)	Flat	Random walks
	ITSS (Tao et al., 2007)	Hierarchical	Semantic similarity
	NtN (Done et al., 2010)	Hierarchical	Singular value decomposition
	dRW (Yu et al., 2015d)	Hierarchical	Random walks
	PILL (Yu et al., 2015b)	Hierarchical	Random walks
	DeepGO (Kulmanov et al., 2017)	Hierarchical	Deep learning
	NewGOA (Yu et al., 2018a)	Hierarchical	Bi-random walks
	AsyRW (Zhao et al., 2019b)	Hierarchical	Bi-random walks
Identifying *noisy* annotations	NoisyGOA (Lu et al., 2016)	Hierarchical	Semantic-based kNN
	NoGOA (Yu et al., 2017c)	Hierarchical	Sparse representation
	NFA (Lu et al., 2018)	Hierarchical	Sparse representation
Selecting *negative* annotations	ALBias (Youngs et al., 2013)	Flat	Bayesian model
	ProPN (Fu et al., 2016b)	Flat	Random walks
	SNOB (Youngs et al., 2014)	Hierarchical	Bayesian model
	NETL (Youngs et al., 2014)	Hierarchical	Topic model
	IFDR (Yu et al., 2017b)	Hierarchical	Semi-supervised linear regression
	NegGOA (Fu et al., 2016a)	Hierarchical	Random walks

#### 3.1.1. Flat Inter-Relations-Based Solutions

Early solutions simply treated gene function prediction as a binary (or multi-class) classification problem (Hua and Sun, 2001; Lanckriet et al., 2003; Leslie et al., 2004). These solutions accounted for neither the flat nor the hierarchical inter-relations between GO terms. As a result, they are generally less accurate than more advanced solutions (Tao et al., 2007; Pandey et al., 2009; Done et al., 2010; Liu et al., 2016), which take into account the various inter-relation among GO terms.

To predict new GO annotations of genes, Elisseeff and Weston (2002) pioneered a rank-based support vector machine that ranked relevant annotations of genes ahead of irrelevant ones. Yu et al. (2012a) and Zhang et al. (2012) used the empirical co-occurrence of two GO terms annotated to the same genes to predict new annotations of genes, and Yu et al. (2013b, 2015a) further selectively fused multiple functional networks for gene function prediction. To replenish the missing annotations of partially annotated genes, Yu et al. (2012b) proposed a gene function prediction model based on weak label learning (ProWL), in which the labels of the annotated training data were incomplete. ProWL performs the prediction for one GO term at a time. To solve this problem, Yu et al. (2013a) presented an algorithm called ProDM, which uses the maximized dependency between the features and GO annotations of genes to predict missing (or new) GO annotations of genes. Chicco et al. (2014) took advantage of the equivalence between a truncated singular value decomposition and an autoencoder neural network, and employed an autoencoder on the gene-term association matrix to predict missing annotations of genes.

To identify negative examples (or negative annotations with respect to a GO term/gene), some models (Mostafavi and Morris, 2009; Cesa-Bianchi et al., 2012) utilized heuristics to determine negative examples first and, thus, reduce the impact of an absence of negative examples in discriminative learning. Next, these models merged the selected negative examples to make a prediction. For example, Guan et al. (2008) assumed that the negative examples of a given term were all genes not annotated with that term. Mostafavi and Morris (2009) and Cesa-Bianchi et al. (2012) presumed that negative examples of a target term came from the genes which were not annotated with sibling terms of that term. This hypothesis may be often violated, since a gene may be annotated with one or more of those sibling terms as more experimental evidence becomes available. Youngs et al. (2013) introduced a model called ALBias, which assumed that the negative examples of a gene should root in the terms with the smallest probability of being annotated to that gene. The negative examples selected by ALBias can boost the performance of gene function predictions. To take advantage of information about features of genes and the available-but-scanty negative examples, Fu et al. (2016b) proposed a gene function prediction approach using positive and negative examples (ProPN). In ProPN, a signed hybrid directed graph encodes the positive and negative examples, the interactions between genes and the flat inter-relations between terms. Then, label propagation on the graph identifies the negative examples.

Irrespective of the target task, these solutions generally focus on using the co-occurrence of GO terms annotated to the same genes. Although some of them also use the annotations augmented by *True Path Rule*, they still do not explicitly include the important hierarchical inter-relations among the GO terms.

#### 3.1.2. Hierarchical Inter-Relations-Based Solutions

Many models use the hierarchical inter-relations between GO terms and prove that the appropriate use of inter-relations can improve the gene function prediction (Tao et al., 2007; Done et al., 2010; Yu et al., 2015b). For example, Barutcuoglu et al. (2006) organized the predictions obtained from multiple binary classifiers for different terms in a Bayesian network derived from the GO hierarchy. Valentini (2011) and Cesa-Bianchi et al. (2012) further introduced a bi-directional asymmetric flow of information based on the GO hierarchy using an ensemble method, in which the positive predictions for a node propagated to its ancestors in a recursive way, while the negative predictions propagated to its offsprings. Obozinski et al. (2008) focused on calibrating and combining independent predictions to obtain a set of probabilistic predictions that are consistent with the topology of the ontology. Kahanda and Ben-Hur (2017) proposed a structured output solution that adopted a structural kernel function.

King et al. (2003) directly applied the annotation patterns of genes to induce a decision tree or Bayesian classifier to predict gene functions. However, neither classifiers was reliable for sparse GO terms, which are annotated with too few ( ≤ 10) genes. Tao et al. (2007) quantified the semantic similarity between genes by combing the hierarchical relationships between terms and known GO annotations of genes, then using a *k* nearest neighbor (kNN) classifier with the semantic similarity to predict unknown annotations of genes. Pandey et al. (2009) employed Lin's similarity (Lin, 1998) to capture the inter-relations between hierarchically organized terms and to infer annotations of genes. Done et al. (2010) introduced a method called NtN, which applies singular value decomposition (SVD) (Golub and Reinsch, 1971) on the gene-term association matrix, whose entries are weighted by the term frequency-inverse document frequency and GO hierarchy; thus, the semantic relationships between genes and between terms were explored and the missing associations between genes and terms were completed. Yu et al. (2015b) utilized the hierarchical and flat inter-relations among terms to predict additional annotations of partially annotated genes. However, this solution ignored GO terms in the GO hierarchy that were not yet annotated to studied genes. To solve this problem, Yu et al. (2015d) introduced a downward Random Walks model (dRW), which performed random walks on the GO hierarchy while taking the terms annotated to a gene as the initial nodes. Given the structural difference between the GO terms subgraph and the genes subgraph, Yu et al. (2018a) proposed a method called NewGOA, which used a bi-random walk strategy on a hybrid graph to predict new annotations of genes. Zhao et al. (2019b) quantified the individual walk-lengths for each node of a hybrid network composed of genes, GO terms and their hierarchical relations; then, a random walk with individual walk-lengths on the network was performed to achieve cross-species gene function prediction. Kulmanov et al. (2017) developed a deep learning-based approach that utilized the GO structure as background information to optimize the predictions.

To select negative examples, Youngs et al. (2014) proposed two algorithms: selection of negatives through observed bias (SNOB) and negative examples from topic likelihood (NETL). SNOB approximated the empirical conditional probability between terms using both direct and GO-hierarchy augmented annotations. NTEL assumed a gene is a document and all terms affiliated with that gene are words of that document; then it used a Latent Dirichlet Allocation topic model (Blei et al., 2003) to select negative examples. Fu et al. (2016a) proposed a negative GO annotations selection approach (NegGOA) that leveraged GO hierarchy, random walks, and co-occurrence patterns of annotations to select negative examples of a gene. Experimental study has demonstrated that NegGOA suffered less from incomplete annotations than NETL or SNOB, and that the selected negative examples improved the performance of gene function prediction. Yu et al. (2017b) applied a random walk on the GO hierarchy and biological network to enrich the links between nodes, and then factorized the updated relational matrices of hierarchy and the network into two low-rank numeric matrices (one for the feature data matrix and the other for the GO label matrix), and finally imposed a semi-supervised classification on the two low-rank matrices to infer positive or negative annotations of genes.

The GO hierarchical structure has also been used to identify noisy annotations, which is a less-studied but practical topic of gene function prediction. Since GO annotations of genes are collected from different sources (like crowdsourcing), these annotations are inevitably inaccurate (Huntley et al., 2014). Lu et al. (2016) proposed a novel model (NoisyGOA) that measured the taxonomic similarity between ontological terms using the GO hierarchy and the semantic similarity between genes using annotations. Next, NoisyGOA utilized the GO annotations of a gene's neighbors to aggregate annotations of the gene. Then, it takes the positive annotations with the lowest aggregated scores as noisy annotations. However, NoisyGOA does not evaluate the reliability of different annotations, and includes noisy annotations when quantifying the semantic similarity between genes. To address that, Lu et al. (2018) preset weights for different evidence codes and upward-propagated weights to ancestor annotations via the GO hierarchy. Next, they measured the semantic similarity between genes by *l*_1_-norm regularized sparse representation on the weighted gene-term association matrix, and took advantage of annotations of semantic neighbors to identify noisy annotations of a gene. Further, Yu et al. (2017c) introduced a more advanced and adaptive approach (NoGOA), which used evidence codes of annotations to deferentially weight annotations and sparse representation to quantify the similarity between genes to identify noisy annotations.

Overall, these solutions each model GO by using the pattern of GO annotations and/or GO hierarchy. Therefore, they generally obtain a better performance than counterparts without such modeling.

### 3.2. Gene Function Prediction by Compressing Massive GO Terms

GO now includes more than 45,000 GO terms, and most GO annotations of genes are sparse and incomplete. As such, predicting the associations between genes and massive terms is rather difficult. Some solutions (Emmert-Streib and Dehmer, 2009; Li et al., 2009; Yu et al., 2018a) use different techniques to utilize the GO hierarchy graph and to boost performance with respect to sparse GO terms, which are annotated to too few genes. However, they still have to handle massive GO terms. In actual fact, the huge number of GO terms also causes a heavy computation burden for GO-based semantic similarity studies (Mistry and Pavlidis, 2008; Yu et al., 2015d). To alleviate this difficulty, researchers have tried to compress massive terms, and predict gene functions in a compressed label space. Based on the adopted techniques, existing solutions can be divided into two types: (i) *matrix factorization-based* and (ii) *hashing coding-based* techniques. These methods are summarized in Table 2. Obviously, these solutions have some overlaps with the ones introduced in the previous subsections. These solutions demonstrate that compressing GO terms improves accuracy and may even boost efficiency (Wang et al., 2015; Yu et al., 2017e; Zhao et al., 2019a).

**Table 2:** Exemplar solutions based on compressing GO terms.

	**Solutions**	**Inter-relations**
Matrix factorization	ProCMF (Yu et al., 2017d)	Flat
	clusDCA (Wang et al., 2015)	Hierarchical
	NtN (Done et al., 2010)	Hierarchical
	clusDCA (Wang et al., 2015)	Hierarchical
	ProsNet (Wang et al., 2017)	Hierarchical
	IFDR (Yu et al., 2017b)	Hierarchical
	NMFGO (Yu et al., 2020b)	Hierarchical
	ZOMF (Zhao et al., 2019c)	Hierarchical
	LSDRs (Makrodimitris et al., 2019)	Hierarchical
Hash learning	HashGO (Yu et al., 2017e)	Hierarchical
	HPHash (Zhao et al., 2019a)	Hierarchical

#### 3.2.1. Matrix Factorization-Based Solutions

Some efforts have been made toward applying matrix factorization-based solutions to compress sparse GO terms and to infer annotations of genes (Done et al., 2010; Wang et al., 2015; Yu et al., 2017b). NtN (Done et al., 2010) and IFDR (Yu et al., 2017b) are methods already mentioned in section 3.1.2. In addition, Yu et al. (2017d) proposed ProCMF to explore the latent relationships between genes and GO terms by matrix factorization. ProCMF factorized the gene-term association matrix into two low-rank matrices, and then defined two smoothness terms on these two matrices to use multiple functional association networks of genes and flat inter-relations between GO terms. These two terms also guide the matrix factorization and the approximation of the to-be-predicted gene-term association matrix. Wang et al. (2015) introduced a method called clusDCA based on Diffusion Component Analysis (DCA) (Cho et al., 2015). clusDCA individually performed a random walk on the GO DAG and on the biological networks to capture information about the underlying structure, then obtained two updated adjacency matrices. To reduce noise, it applied SVD on the two matrices to compress them into two low-dimensional matrices. After that, clusDCA optimized a relational matrix between low-dimensional matrices to explore the latent relations, and to predict the associations between genes and GO terms. clusDCA manifested a significantly improved performance on sparse terms. Yu et al. (2020b) introduced a method called NMFGO, which combined non-negative matrix factorization (NMF) (Lee and Seung, 1999) with a GO DAG regularization term to factorize the gene-term association matrix into two low-rank matrices. Next, NMFGO used the low-rank matrices to explicitly calculate the semantic similarity between genes. After that, NMFGO predicted the low-rank labels of a gene based on the low-rank labels of its semantic neighbors. Then, it restored the predictions to the original GO terms. Makrodimitris et al. (2019) recently experimentally evaluated a series of label-compression solutions based on matrix factorization and proved that compressed labels can boost the prediction performance.

However, the matrix factorization-based methods above lack interpretability of the compressed labels, and suffer from an inherent problem of thresholding both the relevant and irrelevant GO annotations from the predicted numeric gene-term association matrix. This problem is also found in multi-label learning (Pillai et al., 2013). To solve these problems, Zhao et al. (2019c) introduced a method based on zero-one matrix factorization (ZOMF). ZOMF decomposed the gene-term association matrix into two low-rank matrices with entry values restricted to one or zero, then explored the inner latent relationships between the genes and terms. Next, it defined two smoothness terms on these two low-rank matrices with respect to the gene-gene interactions and the structural relationships between terms, thus guiding the optimization of low-rank matrices. Finally, it reconstructed the association matrix using the optimized two low-rank matrices to predict gene functions. ZOMF did not need to threshold the reconstructed association probability matrix, and the compressed zero-one labels had a more intuitive explanation than compressed labels.

#### 3.2.2. Hashing-Based Solutions

To achieve low storage and fast retrieval, hashing has been widely used in big data applications (Wang et al., 2016; Liu et al., 2019). For example, Tian et al. (2016) used hash tables to store essential information learned from GO DAG and to efficiently compute the semantic similarity of genes. Empirical studies show that hash tables-based solutions can speed up diverse semantic similarity metrics, e.g., the group-based one (Teng et al., 2013) and Best Match Average (Pesquita et al., 2008). Researchers also recently employed hashing learning techniques to convert the typical one-hot coding of massive GO terms into short binary hashing codes. For example, Yu et al. (2017e) adopted a hashing technique that preserved the graph structure from Liu et al. (2011) to represent a large set of GO terms with compact binary codes, and then computed semantic similarity between the genes using the Hamming distance to predict gene functions. However, this method did not obey the GO hierarchy very well. To solve this problem, Zhao et al. (2019a) introduced a hashing method that preserved the ontology hierarchy (HPHash), which sought a set of hash functions to maintain the GO hierarchy order and the taxonomic similarity between the terms. Then, HPHash used the hash functions to compress a high-dimensional gene-term association matrix into a low-dimensional binary matrix, and predicted the gene functions therein. HPHash improved the prediction accuracy, and can be used as a plugin to boost the BLAST-based gene function prediction (Zhang et al., 1997; You et al., 2018).

### 3.3. Cross-Species Solutions

GO is a community-collaborative effort in functional genomics, and GO terms are generally organized in a species-neutral way to reflect the broad domain knowledge of biology. Due to differences in the preferences of biologist and in research ethics for experiments involving humans, animals, and plants, the curated annotations of genes for different species are biased, incomplete, and imbalanced (Schnoes et al., 2013; Dessimoz and Škunca, 2017; Zhao et al., 2019b). Two species with high homology have a large number of homologous genes, which should share similar (or even identical) GO annotations (Schnoes et al., 2013). Unfortunately, contemporary homologous genes are associated with different GO terms, due to the bias of biologists and diverse focuses on different species. Therefore, it is interesting to leverage the shared GO structure and complementary annotations of genes for cross-species gene function prediction.

In the early stages, typical cross-species solutions only involved the sequence data along with BLAST and PSI-BLAST (Zhang et al., 1997), but these solutions were unreliable, and the sequence identification was <25% (Shehu et al., 2016). Eisen (1998) found that utilizing evolutionary information improved gene function prediction. Guided by this observation, some databases based on the phylogenetic trees of animal-gene families appeared, such as TreeFam (Li et al., 2006). Chikina and Troyanskaya (2011) leveraged gene sequence and expression data to identify function analogous genes, and obtained an improved accuracy. However, these solutions ignored GO. To consider GO, Mitrofanova et al. (2011) presented a GO chain-graph-based approach to improve gene function prediction, which utilized high inter-species sequence homology, the PPIs of two or more species together and the GO hierarchy to construct a heterogeneous network. But this inter-species method only considered a small number of GO terms. Park et al. (2013) demonstrated that comparing the sequences of just two genes participating in the same biological processes is somewhat inaccurate. Using other genomic data, such as gene expression, can supplement traditional sequence-similarity measures to boost the performance when evalusting biological-process functions. Some other solutions attempted more advanced sequence or physical-chemical similarity metrics to improve the function prediction (Vidulin et al., 2016; Kulmanov et al., 2017; You et al., 2018; Kulmanov and Hoehndorf, 2020). For example, You et al. (2018) recently presented the GOLabeler, which separately trained five different classifiers from five different feature descriptors on sequence data, and then combined these classifiers to make a prediction. These attempts typically assumed that the annotations of the “well-annotated” species were complete, which is not true (Jiang et al., 2014). Moreover, they neglected the dynamic, mutually supplementary GO annotations of the close-homology species. Yu et al. (2016b) studied cross-species gene function prediction based on semantic similarity. They separately explored the prediction performance for two species with high or low homology, finding that annotations of highly-homologous species were complementary, while those of less homologous species did not complement each other. Kulmanov et al. (2017) developed a deep learning-based method (DeepGO) to predict gene function from sequences. In DeepGO, the deep learning model predicted the GO annotations of genes based on gene sequences and dependencies between GO terms. To leverage the GO annotations of different species, Zhao et al. (2019b) constructed a heterogeneous network including the GO hierarchy, intra- and inter-species subnetworks. Then, they introduced an asynchronous random work on the heterogeneous network to predict gene functions.

### 3.4. GO-Based Semantic-Similarity Measures and Applications

The semantic similarity between genes is quantified using GO annotations and/or GO hierarchy. It is positively correlated with the feature similarity between them, which is computed from other biological data (Pesquita et al., 2009; Yu et al., 2015d). Therefore, semantic-based (and also sequence similarity- or interaction network-based) gene function prediction has been popular in recent years (Tao et al., 2007; Yu et al., 2015d, 2016a, 2017c,e).

Semantic similarity-based methods typically use the semantic similarity to select the neighborhood genes and predict the annotations of a gene based on annotations of those neighborhood genes. ITSS (Tao et al., 2007), dRW (Yu et al., 2015d), HashGO (Yu et al., 2017e), HPHash (Zhao et al., 2019a), and NMFGO (Yu et al., 2020b) are some representative methods introduced in sections 3.1.2, 3.2.2. In addition, the semantic similarity is integrated with other feature similarities for gene function prediction (Yu et al., 2015c, 2016a). For example, Yu et al. (2016a) proposed a semantic data fusion method (SimNet), which optimized the weights of multiple functional association networks to align with a semantic-similarity kernel matrix induced from the GO annotations of genes. After that, SimNet applied these weights to fuse the networks into a composite network, and then performed random walks on the composite network to make a prediction.

Measures of the similarity between genes can be extended from taxonomic similarity measures between GO terms. Existing similarity measures between genes can be further divided into two categories (Pesquita et al., 2009), pairwise and groupwise. Pairwise measures generally employ an average combination (Lord et al., 2003), maximum combination (Sevilla et al., 2005), or best match average combination (BMA) to integrate the proximity between pairwise terms. Among them, BMA provides a good balance between the maximum and average measure, since the latter two measures are inherently influenced by the number of terms being combined (Pesquita et al., 2009). Groupwise measures directly apply set (Mistry and Pavlidis, 2008), graph (Pesquita et al., 2008; Teng et al., 2013), or vector operator to compute the similarity between two sets of terms. For example, Mistry and Pavlidis (2008) introduced a set based metric called term overlap (TO), which takes into account the ratio between the number of shared annotations and minimum number of annotations of two genes. Graph-based measures organize terms annotated to a gene by a subgraph of DAG and then use graph comparing techniques to quantify the similarity between genes, i.e., simGIC (Pesquita et al., 2008) and SORA (Teng et al., 2013). The associations between a gene and all its terms can be encoded as a binary vector; vector-based measures then directly calculate the similarity between genes on the binary vectors using traditional similarity metrics (i.e., cosine and Hamming distances). The methods mentioned above use only the GO annotations and structure, whereas Peng et al. (2018) presented a similarity measure that integrated information from gene co-function networks, the GO structure and annotations.

To facilitate effective exploration of these semantic measures, some online tools or packages have been developed for the community. Yu et al. (2010) introduced an R package called GOSemSim to efficiently compute the semantic similarity between individual GO terms, sets of GO terms, genes or gene clusters. Peng et al. (2016) developed a web tool called InteGO2 to select the most appropriate measure from a set of measures using a voting method, or to integrate measures via a meta-heuristic search method. Mazandu et al. (2015) introduced a Python portable application called A-DaGO-Fun, which assembled diverse semantic measures and biological applications using these measures.

However, most solutions based on semantic similarity are still impacted by incomplete GO annotations. For a gene without any GO annotations, its semantic similarity with other genes is zero. Another limitation of semantic similarity-based solutions is that they cannot predict new annotations for a gene without any annotations. Furthermore, semantic measures are computed with respect to massive GO terms and, thus, are less reliable with sparse annotations. To address the last issue, some efforts have been made toward compressing these terms before measuring the semantic similarity (Done et al., 2010; Yu et al., 2017e, 2020b; Zhao et al., 2019a); these were reviewed in previous subsections.

## 4. Remaining Challenges and Potential Topics

Despite much progress, the intrinsic complexity of GO-based gene function prediction, the evolution of GO and the importance of reliable GO annotations for various domains mean that there are still interesting and challenging research directions, which deserve further efforts.

First, the GO annotations of genes are still incomplete, shallow, imbalanced across species and even noisy (Thomas et al., 2012; Dessimoz and Škunca, 2017). Since the semantic similarity between genes may not faithfully reflect the actual similarity between genes or terms with incomplete annotations, semantic similarity-based solutions can only be applied for species with sufficient annotations. Although several semantic similarity-based solutions make specific use of the GO hierarchy, GO annotations (Tao et al., 2007; Done et al., 2010; Xu et al., 2013; Yu et al., 2015b,d) and additional data sources (Peng et al., 2018; Yu et al., 2020b) to obtain an improved performance, they are mostly based on the assumption of complete annotations. In addition, many solutions suffer from an overwhelming computational load when handling massive GO terms. Hence, more efficient and effective models are still welcomed.

Second, for massive GO terms, the models based on compressed GO terms (Done et al., 2010; Wang et al., 2015; Yu et al., 2017e, 2020b; Zhao et al., 2019a) have attracted increasing interest. Although the compressed labels allow researchers to explore and employ potential relationships between terms, more theoretically sound label-compression solutions, which enable efficient gene function prediction with improved efficiency and reliability, are still anticipated.

Third, multi-omics data can reflect gene function from different aspects and they complement each other. Some efforts have been made to combine GO and heterogeneous proteomics/genomics data (Cho et al., 2016; Yu et al., 2016a, 2017d), but they often suffer from a large number of GO terms. Therefore, they have to project heterogeneous data onto the common latent feature space, which obscures the intrinsic structures of the respective data sources. More advanced integrative solutions must integrate these heterogeneous biological data and the GO knowledge more effectively.

Fourth, due to the research priorities of biologists and animal/plant ethics, the collected GO annotations of genes are imbalanced across different species (Schnoes et al., 2013). Many species have scarce annotations, and their annotations must be electronically inferred from those of relatively well-annotated species. Some studies show that the GO annotations of homologous genes across species are complementary. One fruitful direction would be to credibly transfer annotations from several well-annotated and curated species to less-studied species.

Fifth, most existing solutions focus on predicting the new annotations of a newly-sequenced gene or the missing annotations of a gene with sparse annotations. In fact, gene function prediction relies on the known positive and negative annotations of a gene, but conventionally only the positive annotations of genes are reported and, thus, recorded in GO. Therefore, it lacks negative annotations, which limits the discriminative ability of function prediction models (Youngs et al., 2014; Fu et al., 2016a). Noisy annotations are also still largely overlooked by the community, which may mislead wet-lab experimental verification, GO enrichment analysis, and more. More efforts can be devoted into identifying noisy annotations and irrelevant (or negative) annotations of genes.

Last but not least, beside proteins, other gene products like miRNAs and lncRNAs also play important roles in many life processes and have associations with different complex diseases (Lu et al., 2008; Chen et al., 2012; Deng et al., 2019; Zou et al., 2019). Our preliminary studies (Yu et al., 2017a, 2018b; Fu et al., 2018; Wang et al., 2019) show that using GO appropriately can boost the prediction of lncRNA-disease associations, and GO has some overlaps with Disease Ontology (Schriml et al., 2011), which also adopts a DAG to hierarchically organize disease terms. For example, GO has been used to find functional similarities in genes that are overexpressed or underexpressed in diseases (Chen et al., 2013), and our empirical results showed that the exclusion of GO annotations of genes significantly compromised the precision of an lncRNA-disease association prediction (Yu et al., 2017a; Fu et al., 2018). Another issue is that alternative splicing causes a gene to be translated into different isoforms or protein variants, but GO collectively stores the associations between GO terms and genes irrespective these variants. Differentiating the GO annotations of individual isoforms can provide a deeper analysis of living processes (Li et al., 2014). Our recent study confirmed that considering the GO hierarchy also helps to identify the functions of individual isoforms (Wang et al., 2020; Yu et al., 2020a). The accumulated experiences of using GO for gene function prediction are expected to shed light on the predicted functions of other molecules (i.e., ncRNAs).

## 5. Conclusions

Identifying the functional roles of gene products such as proteins and RNAs is one of the fundamental tasks in the post-genomic era. Given the incomplete functional knowledge of genes, we have to admit that existing gene function prediction solutions are still no substitute for wet-lab experiments. Rather, they are an important supplementary technique. As more evidence of gene functions is accumulated from experiments, the gene function prediction solutions will become more competent.

Our survey reviews the literature of ongoing studies of gene function prediction using GO, with the aim of expediting research into reliable gene function prediction. We may neglect some important work related to GO-based computational gene function prediction, given multiplicity and diverse progress in various areas. The main challenges of gene function prediction are: (i) GO annotations that are incomplete, sparse, shallow, and imbalanced within and between species; (ii) massive structurally organized GO terms; and (iii) increasing relevant and irrelevant multi-type biological data. In summary, although various computational methods based on GO have been proposed, there are still promising topics and challenges that deserve further efforts.

## Author Contributions

YZ and GY drafted the manuscript. MG and GY conceived the whole program, extensively revised the manuscript, and finally approved the final manuscript. JW, JC, and XZ participated in the discussion and revision of this manuscript.

## Conflict of Interest

The authors declare that the research was conducted in the absence of any commercial or financial relationships that could be construed as a potential conflict of interest.

## Footnotes

^1^webofknowledge.com

## References

[B1] Ashburner M. Ball C. A. Blake J. A. Botstein D. Butler H. Cherry J. M.. (2000). Gene ontology: tool for the unification of biology. Nat. Genet. 25, 25–29. 10.1038/75556 10802651 PMC3037419

[B2] Barabási A.-L. Gulbahce N. Loscalzo J. (2011). Network medicine: a network-based approach to human disease. Nat. Rev. Genet. 12, 56–68. 10.1038/nrg2918 21164525 PMC3140052

[B3] Barutcuoglu Z. Schapire R. E. Troyanskaya O. G. (2006). Hierarchical multi-label prediction of gene function. Bioinformatics 22, 830–836. 10.1093/bioinformatics/btk048 16410319

[B4] Blake J. A. (2013). Ten quick tips for using the gene ontology. PLoS Comput. Biol. 9:e1003343. 10.1371/journal.pcbi.1003343 24244145 PMC3828149

[B5] Blei D. M. Ng A. Y. Jordan M. I. (2003). Latent dirichlet allocation. J. Mach. Learn. Res. 3, 993–1022. 10.1162/jmlr.2003.3.4-5.993

[B6] Buza T. J. (2008). Gene ontology annotation quality analysis in model eukaryotes. Nucleic Acids Res. 36:e12. 10.1093/nar/gkm1167 18187504 PMC2241866

[B7] Cesa-Bianchi N. Re M. Valentini G. (2012). Synergy of multi-label hierarchical ensembles, data fusion, and cost-sensitive methods for gene functional inference. Mach. Learn. 88, 209–241. 10.1007/s10994-011-5271-6

[B8] Chen G. Wang Z. Wang D. Qiu C. Liu M. Chen X.. (2012). LncRNAdisease: a database for long-non-coding RNA-associated diseases. Nucleic Acids Res. 41, D983?D986. 10.1093/nar/gks1099 23175614 PMC3531173

[B9] Chen W.-H. Zhao X.-M. van Noort V. Bork P. (2013). Human monogenic disease genes have frequently functionally redundant paralogs. PLoS Comput. Biol. 9:e1003073. 10.1371/journal.pcbi.1003073 23696728 PMC3656685

[B10] Chicco D. Sadowski P. Baldi P. (2014). “Deep autoencoder neural networks for gene ontology annotation predictions?” in Proceedings of the 5th ACM Conference on Bioinformatics, Computational Biology, and Health Informatics (Newport Beach, CA), 533–540. 10.1145/2649387.2649442

[B11] Chikina M. D. Troyanskaya O. G. (2011). Accurate quantification of functional analogy among close homologs. PLoS Comput. Biol. 7:e1001074. 10.1371/journal.pcbi.1001074 21304936 PMC3033368

[B12] Cho H. Berger B. Peng J. (2015). “Diffusion component analysis: unraveling functional topology in biological networks?” in International Conference on Research in Computational Molecular Biology (Warsaw), 62–64. 10.1007/978-3-319-16706-0_9 PMC5524124 28748230

[B13] Cho H. Berger B. Peng J. (2016). Compact integration of multi-network topology for functional analysis of genes. Cell Syst. 3:540. 10.1016/j.cels.2016.10.017 27889536 PMC5225290

[B14] Clark W. T. Radivojac P. (2011). Analysis of protein function and its prediction from amino acid sequence. Proteins 79, 2086–2096. 10.1002/prot.23029 21671271

[B15] Deng L. Wang J. Zhang J. (2019). Predicting gene ontology function of human micrornas by integrating multiple networks. Front. Genet. 10:3. 10.3389/fgene.2019.00003 30761178 PMC6361788

[B16] Dessimoz C. Skunca N. (2017). The gene ontology handbook. Methods Mol. Biol. 1446,3–68. 10.1007/978-1-4939-3743-1 27812933 PMC6377150

[B17] Done B. Khatri P. Done A. Draghici S. (2010). Predicting novel human gene ontology annotations using semantic analysis. IEEE/ACM Trans. Comput. Biol. Bioinformatics 7, 91–99. 10.1109/TCBB.2008.29 20150671 PMC3712327

[B18] Eisen J. A. (1998). Phylogenomics: improving functional predictions for uncharacterized genes by evolutionary analysis. Genome Res. 8, 163–167. 10.1101/gr.8.3.163 9521918

[B19] Elisseeff A. Weston J. (2002). “A kernel method for multi-labelled classification?” in Advances in Neural Information Processing Systems (Vancouver, BC), 681–687.

[B20] Emmert-Streib F. Dehmer M. (2009). Predicting cell cycle regulated genes by causal interactions. PLoS ONE 4:e6633. 10.1371/journal.pone.0006633 19688096 PMC2723924

[B21] Fu G. Wang J. Domeniconi C. Yu G. (2018). Matrix factorization-based data fusion for the prediction of lncRNA-disease associations. Bioinformatics 34, 1529–1537. 10.1093/bioinformatics/btx794 29228285

[B22] Fu G. Wang J. Yang B. Yu G. (2016a). NegGOA: Negative go annotations selection using ontology structure. Bioinformatics 32, 2996–3004. 10.1093/bioinformatics/btw366 27318205

[B23] Fu G. Yu G. Wang J. Maozu G. (2016b). Protein function prediction using positive and negative example. J. Comput. Res. Dev. 53, 1753–1765. 10.7544/issn1000-1239.2016.20160196

[B24] Gibaja E. Ventura S. (2015). A tutorial on multilabel learning. ACM Comput. Surveys 47:52 10.1145/2716262

[B25] Golub G. H. Reinsch C. (1971). “Singular value decomposition and least squares solutions?”, in Handbook for Automatic Computation. Die Grundlehren der mathematischen Wissenschaften (in Einzeldarstellungen mit besonderer Bercksichtigung der Anwendungsgebiete), Vol. 186, eds F. L. Bauer, A. S. Householder, F. W. J. Olver, H. Rutishauser, K. Samelson, and E. Stiefel (Berlin; Heidelberg: Springer), 134–151. 10.1007/978-3-662-39778-7_10

[B26] Gross A. Hartung M. Kirsten T. Rahm E. (2009). 11Estimating the quality of ontology-based annotations by considering evolutionary changes?” in International Workshop on Data Integration in the Life Sciences (Manchester), 71–87. 10.1007/978-3-642-02879-3_7

[B27] Guan Y. Myers C. L. Hess D. C. Barutcuoglu Z. Caudy A. A. Troyanskaya O. G. (2008). Predicting gene function in a hierarchical context with an ensemble of classifiers. Genome Biol. 9:S3. 10.1186/gb-2008-9-s1-s3 18613947 PMC2447537

[B28] Hua S. Sun Z. (2001). Support vector machine approach for protein subcellular localization prediction. Bioinformatics 17, 721–728. 10.1093/bioinformatics/17.8.721 11524373

[B29] Huntley R. P. Sawford T. Martin M. J. Donovan C. (2014). Understanding how and why the gene ontology and its annotations evolve: the go within uniprot. GigaScience 3, 2047–217X. 10.1186/2047-217X-3-4 24641996 PMC3995153

[B30] Hvidsten T. R. Komorowski J. Sandvik A. K. Laegreid A. (2001). Predicting gene function from gene expressions and ontologies,? in Pacific Symposium on Biocomputing (Hawaii: World Scientific), 299–310. 10.1142/9789814447362_0030 11262949

[B31] Jiang Y. Clark W. T. Friedberg I. Radivojac P. (2014). The impact of incomplete knowledge on the evaluation of protein function prediction: a structured-output learning perspective. Bioinformatics 30, i609?i616. 10.1093/bioinformatics/btu472 25161254 PMC4147924

[B32] Jiang Y. Oron T. R. Clark W. T. Bankapur A. R. D'Andrea D. Lepore R.. (2016). An expanded evaluation of protein function prediction methods shows an improvement in accuracy. Genome Biol. 17:184. 10.1186/s13059-016-1037-6 27604469 PMC5015320

[B33] Jones C. E. Brown A. L. Baumann A. U. (2007). Estimating the annotation error rate of curated go database sequence annotations. BMC Bioinformatics 8:170. 10.1186/1471-2105-8-170 17519041 PMC1892569

[B34] Kahanda I. Ben-Hur A. (2017). “Gostruct 2.0: Automated protein function prediction for annotated proteins?” in Proceedings of the 8th ACM International Conference on Bioinformatics, Computational Biology, and Health Informatics (Boston, MA), 60–66. 10.1145/3107411.3107417

[B35] Karaoz U. Murali T. Letovsky S. Zheng Y. Ding C. Cantor C. R.. (2004). Whole-genome annotation by using evidence integration in functional-linkage networks. Proc. Natl. Acad. Sci. U.S.A. 101, 2888–2893. 10.1073/pnas.0307326101 14981259 PMC365715

[B36] King O. D. Foulger R. E. Dwight S. S. White J. V. Roth F. P. (2003). Predicting gene function from patterns of annotation. Genome Res. 13, 896–904. 10.1101/gr.440803 12695322 PMC430892

[B37] Kissa M. Tsatsaronis G. Schroeder M. (2015). Prediction of drug gene associations via ontological profile similarity with application to drug repositioning. Methods 74, 71–82. 10.1016/j.ymeth.2014.11.017 25498216

[B38] Kulmanov M. Hoehndorf R. (2020). Deepgoplus: improved protein function prediction from sequence. Bioinformatics 36, 422–429. 10.1101/615260 31350877 PMC9883727

[B39] Kulmanov M. Khan M. A. Hoehndorf R. (2017). DeepGO: predicting protein functions from sequence and interactions using a deep ontology-aware classifier. Bioinformatics 34, 660–668. 10.1093/bioinformatics/btx624 29028931 PMC5860606

[B40] Lanckriet G. R. Deng M. Cristianini N. Jordan M. I. Noble W. S. (2003). “Kernel-based data fusion and its application to protein function prediction in yeast?” in Pacific Symposium on Biocomputing (Hawaii: World Scientific), 300–311. 10.1142/9789812704856_0029 14992512

[B41] Lee D. D. Seung H. S. (1999). Learning the parts of objects by non-negative matrix factorization. Nature 401, 788–791. 10.1038/44565 10548103

[B42] Leslie C. S. Eskin E. Cohen A. Weston J. Noble W. S. (2004). Mismatch string kernels for discriminative protein classification. Bioinformatics 20, 467–476. 10.1093/bioinformatics/btg431 14990442

[B43] Li H. Coghlan A. Ruan J. Coin L. J. Heriche J.-K. Osmotherly L.. (2006). TreeFam: a curated database of phylogenetic trees of animal gene families. Nucleic Acids Res. 34(Suppl. 1), D572?D580. 10.1093/nar/gkj118 16381935 PMC1347480

[B44] Li H.-D. Menon R. Omenn G. S. Guan Y. (2014). The emerging Era of genomic data integration for analyzing splice isoform function. Trends Genet. 30, 340–347. 10.1016/j.tig.2014.05.005 24951248 PMC4112133

[B45] Li X. Chen H. Li J. Zhang Z. (2009). Gene function prediction with gene interaction networks: a context graph kernel approach. IEEE Trans. Inform. Technol. Biomed. 14, 119–128. 10.1109/TITB.2009.2033116 19789115

[B46] Lin D. (1998). “An information-theoretic definition of similarity?” in Proceedings of 15th International Conference on Machine Learning (Madison, WI), 296–304.

[B47] Liu J. Wang J. Yu G. (2016). Protein function prediction by random walks on a hybrid graph. Curr. Proteomics 13, 130–142. 10.2174/157016461302160514004307

[B48] Liu W. Wang J. Kumar S. Chang S.-F. (2011). “Hashing with graphs?” in Proceedings of the 28th International Conference on Machine Learning (Bellevue, WA), 1–8.

[B49] Liu X. Yu G. Domeniconi C. Wang J. Ren Y. Guo M. (2019). “Ranking-based deep cross-modal hashing?” in Proceedings of the AAAI Conference on Artificial Intelligence, Vol. 33 (Hawaii), 4400–4407. 10.1609/aaai.v33i01.33014400

[B50] Lord P. W. Stevens R. D. Brass A. Goble C. A. (2003). Investigating semantic similarity measures across the gene ontology: the relationship between sequence and annotation. Bioinformatics 19, 1275–1283. 10.1093/bioinformatics/btg153 12835272

[B51] Lu C. Chen X. Wang J. Yu G. Yu Z. (2018). Identifying noisy functional annotations of proteins using sparse semantic similarity. Sci. Sin. Inform. 48, 1035–1050. 10.1360/N112017-00105

[B52] Lu C. Wang J. Zhang Z. Yang P. Yu G. (2016). NoisyGOA: Noisy GO annotations prediction using taxonomic and semantic similarity. Comput. Biol. Chem. 65, 203–211. 10.1016/j.compbiolchem.2016.09.005 27670689

[B53] Lu M. Zhang Q. Deng M. Miao J. Guo Y. Gao W.. (2008). An analysis of human microRNA and disease associations. PLoS ONE 3:e3420. 10.1371/journal.pone.0003420 18923704 PMC2559869

[B54] Makrodimitris S. van Ham R. C. Reinders M. J. (2019). Improving protein function prediction using protein sequence and GO-term similarities. Bioinformatics 35, 1116–1124. 10.1093/bioinformatics/bty751 30169569 PMC6449755

[B55] Mazandu G. K. Chimusa E. R. Mbiyavanga M. Mulder N. J. (2015). A-DaGO-Fun: an adaptable gene ontology semantic similarity-based functional analysis tool. Bioinformatics 32, 477–479. 10.1093/bioinformatics/btv590 26476781 PMC5006308

[B56] Mazandu G. K. Chimusa E. R. Mulder N. J. (2016). Gene ontology semantic similarity tools: survey on features and challenges for biological knowledge discovery. Brief. Bioinformatics 18, 886–901. 10.1093/bib/bbw067 27473066

[B57] Mi H. Muruganujan A. Casagrande J. T. Thomas P. D. (2013). Large-scale gene function analysis with the panther classification system. Nat. Protoc. 8, 1551–1566. 10.1038/nprot.2013.092 23868073 PMC6519453

[B58] Mistry M. Pavlidis P. (2008). Gene ontology term overlap as a measure of gene functional similarity. BMC Bioinformatics 9:327. 10.1186/1471-2105-9-327 18680592 PMC2518162

[B59] Mitrofanova A. Pavlovic V. Mishra B. (2011). Prediction of protein functions with gene ontology and interspecies protein homology data. IEEE/ACM Trans. Comput. Biol. Bioinformatics 8, 775–784. 10.1109/TCBB.2010.15 21393654

[B60] Mostafavi S. Morris Q. (2009). “Using the gene ontology hierarchy when predicting gene function?” in Conference on Uncertainty in Artificial Intelligence (Montreal, QC), 419–427.

[B61] Mostafavi S. Morris Q. (2010). Fast integration of heterogeneous data sources for predicting gene function with limited annotation. Bioinformatics 26, 1759–1765. 10.1093/bioinformatics/btq262 20507895 PMC2894508

[B62] Mostafavi S. Ray D. Wardefarley D. Grouios C. Morris Q. (2008). Genemania: a real-time multiple association network integration algorithm for predicting gene function. Genome Biol. 9(Suppl. 1), 1–15. 10.1186/gb-2008-9-s1-s4 18613948 PMC2447538

[B63] Obozinski G. Lanckriet G. Grant C. Jordan M. I. Noble W. S. (2008). Consistent probabilistic outputs for protein function prediction. Genome Biol. 9:S6. 10.1186/gb-2008-9-s1-s6 18613950 PMC2447540

[B64] Pandey G. Kumar V. Steinbach M. (2006). Computational Approaches for Protein Function Prediction: A Survey. Twin Cities: Department of Computer Science and Engineering; University of Minnesota.

[B65] Pandey G. Myers C. L. Kumar V. (2009). Incorporating functional inter-relationships into protein function prediction algorithms. BMC Bioinformatics 10:142. 10.1186/1471-2105-10-142 19435516 PMC2693438

[B66] Park C. Y. Wong A. K. Greene C. S. Rowland J. Guan Y. Bongo L. A.. (2013). Functional knowledge transfer for high-accuracy prediction of under-studied biological processes. PLoS Comput. Biol. 9:e1002957. 10.1371/journal.pcbi.1002957 23516347 PMC3597527

[B67] Pe na-Castillo L. Tasan M. Myers C. L. Lee H. Joshi T. Zhang C. (2008). A critical assessment of *Mus musculus* gene function prediction using integrated genomic evidence. Genome Biol. 9:S2 10.1186/gb-2008-9-s1-s2 PMC2447536 18613946

[B68] Peng J. Li H. Liu Y. Juan L. Jiang Q. Wang Y.. (2016). InteGO2: a web tool for measuring and visualizing gene semantic similarities using gene ontology. BMC Genomics 17:553. 10.1186/s12864-016-2828-6 27586009 PMC5009821

[B69] Peng J. Zhang X. Hui W. Lu J. Li Q. Liu S.. (2018). Improving the measurement of semantic similarity by combining gene ontology and co-functional network: a random walk based approach. BMC Syst. Biol. 12:18. 10.1186/s12918-018-0539-0 29560823 PMC5861498

[B70] Pesquita C. Faria D. Bastos H. Ferreira A. E. Falc ao A. O. Couto F. M. (2008). Metrics for GO based protein semantic similarity: a systematic evaluation. BMC Bioinformatics 9:S4. 10.1186/1471-2105-9-S5-S4 18460186 PMC2367622

[B71] Pesquita C. Faria D. Falcao A. O. Lord P. Couto F. M. (2009). Semantic similarity in biomedical ontologies. PLoS Comput. Biol. 5:e1000443. 10.1371/journal.pcbi.1000443 19649320 PMC2712090

[B72] Pillai I. Fumera G. Roli F. (2013). Threshold optimisation for multi-label classifiers. Pattern Recogn. 46, 2055–2065. 10.1016/j.patcog.2013.01.012

[B73] Radivojac P. Clark W. T. Oron T. R. Schnoes A. M. Wittkop T. Sokolov A.. (2013). A large-scale evaluation of computational protein function prediction. Nat. Methods 10, 221–227. 10.1038/nmeth.2340 23353650 PMC3584181

[B74] Raychaudhuri S. Chang J. T. Sutphin P. D. Altman R. B. (2002). Associating genes with gene ontology codes using a maximum entropy analysis of biomedical literature. Genome Res. 12, 203–214. 10.1101/gr.199701 11779846 PMC155261

[B75] Rhee S. Y. Wood V. Dolinski K. Draghici S. (2008). Use and misuse of the gene ontology annotations. Nat. Rev. Genet. 9, 509–515. 10.1038/nrg2363 18475267

[B76] Ruepp A. Zollner A. Maier D. Albermann K. Hani J. Mokrejs M.. (2004). The funcat, a functional annotation scheme for systematic classification of proteins from whole genomes. Nucleic Acids Res. 32, 5539–5545. 10.1093/nar/gkh894 15486203 PMC524302

[B77] Schnoes A. M. Ream D. C. Thorman A. W. Babbitt P. C. Friedberg I. (2013). Biases in the experimental annotations of protein function and their effect on our understanding of protein function space. PLoS Comput. Biol. 9:e1003063. 10.1371/journal.pcbi.1003063 23737737 PMC3667760

[B78] Schriml L. M. Arze C. Nadendla S. Chang Y.-W. W. Mazaitis M. Felix V.. (2011). Disease ontology: a backbone for disease semantic integration. Nucleic Acids Res. 40, D940?D946. 10.1093/nar/gkr972 22080554 PMC3245088

[B79] Schug J. Diskin S. Mazzarelli J. Brunk B. P. Stoeckert C. J. (2002). Predicting gene ontology functions from ProDom and CDD protein domains. Genome Res. 12, 648–655. 10.1101/gr.222902 11932249 PMC187511

[B80] Schwikowski B. Uetz P. Fields S. (2000). A network of protein-protein interactions in yeast. Nat. Biotechnol. 18, 1257–1261. 10.1038/82360 11101803

[B81] Sevilla J. L. Segura V. Podhorski A. Guruceaga E. Mato J. M. Martinez-Cruz L. A.. (2005). Correlation between gene expression and GO semantic similarity. IEEE/ACM Trans. Comput. Biol. Bioinformatics 2, 330–338. 10.1109/TCBB.2005.50 17044170

[B82] Shehu A. Barbar D. Molloy K. (2016). “A survey of computational methods for protein function prediction?”, in Big Data Analytics in Genomics, ed K. C. Wong (Cham: Springer), 225–298. 10.1007/978-3-319-41279-5_7

[B83] Tao Y. Li J. Friedman C. Lussier Y. A. (2007). Information theory applied to the sparse gene ontology annotation network to predict novel gene function. Bioinformatics 23, i529?i538. 10.1093/bioinformatics/btm195 17646340 PMC2882681

[B84] Teng Z. Guo M. Liu X. Dai Q. Wang C. Xuan P. (2013). Measuring gene functional similarity based on group-wise comparison of go terms. Bioinformatics 29, 1424–1432. 10.1093/bioinformatics/btt160 23572412

[B85] The Gene Ontology Consortium (2017). Expansion of the gene ontology knowledgebase and resources. Nucleic Acids Res. 45, D331?D338 10.1093/nar/gkw1108 27899567 PMC5210579

[B86] Thomas P. D. Mi H. Lewis S. (2007). Ontology annotation: mapping genomic regions to biological function. Curr. Opin. Chem. Biol. 11, 4–11. 10.1016/j.cbpa.2006.11.039 17208035

[B87] Thomas P. D. Wood V. Mungall C. J. Lewis S. E. Blake J. A. (2012). On the use of gene ontology annotations to assess functional similarity among orthologs and paralogs: a short report. PLoS Comput. Biol. 8:e1002386. 10.1371/journal.pcbi.1002386 22359495 PMC3280971

[B88] Tian Z. Wang C. Guo M. Liu X. Teng Z. (2016). SGFSC: speeding the gene functional similarity calculation based on hash tables. BMC Bioinformatics 17:445. 10.1186/s12859-016-1294-0 27814675 PMC5096311

[B89] Tiwari A. K. Srivastava R. (2014). A survey of computational intelligence techniques in protein function prediction. Int. J. Proteomics 2014:845479. 10.1155/2014/845479 25574395 PMC4276698

[B90] Troyanskaya O. G. Dolinski K. Owen A. B. Altman R. B. Botstein D. (2003). A bayesian framework for combining heterogeneous data sources for gene function prediction (in *Saccharomyces cerevisiae*). Proc. Natl. Acad. Sci. U.S.A. 100, 8348–8353. 10.1073/pnas.0832373100 12826619 PMC166232

[B91] Valentini G. (2011). True path rule hierarchical ensembles for genome-wide gene function prediction. IEEE/ACM Trans. Comput. Biol. Bioinformatics 8, 832–847. 10.1109/TCBB.2010.38 20479498

[B92] Valentini G. (2014). Hierarchical ensemble methods for protein function prediction. ISRN Bioinformatics 2014:901419. 10.1155/2014/901419 25937954 PMC4393075

[B93] Vidulin V. Šmuc T. Supek F. (2016). Extensive complementarity between gene function prediction methods. Bioinformatics 32, 3645–3653. 10.1093/bioinformatics/btw532 27522084

[B94] Wang J. Liu W. Kumar S. Chang S. F. (2016). Learning to hash for indexing big data - a survey. Proc. IEEE 104, 34–57. 10.1109/JPROC.2015.2487976

[B95] Wang K. Wang J. Domeniconi C. Zhang X. Yu G. (2020). Isoform function prediction based on bi-random walks on a heterogeneous network. Bioinformatics 36, 1864–1871. 31250882 10.1093/bioinformatics/btz535

[B96] Wang S. Cho H. Zhai C. Berger B. Peng J. (2015). Exploiting ontology graph for predicting sparsely annotated gene function. Bioinformatics 31, i357?i364. 10.1093/bioinformatics/btv260 26072504 PMC4542782

[B97] Wang S. Qu M. Peng J. (2017). “ProSNet: Integrating homology with molecular networks for protein function prediction?” in Pacific Symposium on Biocomputing (Hawaii), 27–38. 10.1142/9789813207813_0004 PMC5319591 27896959

[B98] Wang Y. Yu G. Domeniconi C. Wang J. Zhang X. Guo M. (2019). Selective matrix factorization for multi-relational data fusion,? in International Conference on Database Systems for Advanced Applications, 313–329. 10.1007/978-3-030-18576-3_19

[B99] Xu Y. Guo M. Shi W. Liu X. Wang C. (2013). A novel insight into gene ontology semantic similarity. Genomics 101, 368–375. 10.1016/j.ygeno.2013.04.010 23628645

[B100] Xuan P. Sun C. Zhang T. Ye Y. Shen T. Dong Y. (2019). A gradient boosting decision tree-based method for predicting interactions between target genes and drugs. Front. Genet, 10:459. 10.3389/fgene.2019.00459 31214240 PMC6555260

[B101] You R. Zhang Z. Xiong Y. Sun F. Mamitsuka H. Zhu S. (2018). GOLabeler: Improving sequence-based large-scale protein function prediction by learning to rank. Bioinformatics 34, 2465–2473. 10.1093/bioinformatics/bty130 29522145

[B102] Youngs N. Penfold-Brown D. Bonneau R. Shasha D. (2014). Negative example selection for protein function prediction: the NoGo database. PLoS Comput. Biol. 10:e1003644. 10.1371/journal.pcbi.1003644 24922051 PMC4055410

[B103] Youngs N. Penfold-Brown D. Drew K. Shasha D. Bonneau R. (2013). Parametric Bayesian priors and better choice of negative examples improve protein function prediction. Bioinformatics 29, 1190–1198. 10.1093/bioinformatics/btt110 23511543 PMC3634187

[B104] Yu G. Domeniconi C. Rangwala H. Zhang G. (2013a). “Protein function prediction using dependence maximization?” in Joint European Conference on Machine Learning and Knowledge Discovery in Databases (Prague: Springer), 574–589. 10.1007/978-3-642-40988-2_37

[B105] Yu G. Domeniconi C. Rangwala H. Zhang G. Yu Z. (2012a). “Transductive multi-label ensemble classification for protein function prediction?” in Proceedings of the 18th ACM SIGKDD International Conference on Knowledge Discovery and Data Mining (Beijing), 1077–1085. 10.1145/2339530.2339700

[B106] Yu G. Fu G. Lu C. Ren Y. Wang J. (2017a). BRWLDA: bi-random walks for predicting lncRNA-disease associations. Oncotarget 8:60429. 10.18632/oncotarget.19588 28947982 PMC5601150

[B107] Yu G. Fu G. Wang J. Guo M. (2017b). Predicting irrelevant functions of proteins based on dimensionality reduction. Sci. Sin. Inform. 47, 1349–1368. 10.1360/N112017-00009

[B108] Yu G. Fu G. Wang J. Zhao Y. (2018a). NewGOA: Predicting new go annotations of proteins by bi-random walks on a hybrid graph. IEEE/ACM Trans. Comput. Biol. Bioinformatics 15, 1390–1402. 10.1109/TCBB.2017.2715842 28641268

[B109] Yu G. Fu G. Wang J. Zhu H. (2016a). Predicting protein function via semantic integration of multiple networks. IEEE/ACM Trans. Comput. Biol. Bioinformatics 13, 220–232. 10.1109/TCBB.2015.2459713 26800544

[B110] Yu G. Li F. Qin Y. Bo X. Wu Y. Wang S. (2010). GOSemSim: an R package for measuring semantic similarity among go terms and gene products. Bioinformatics 26, 976–978. 10.1093/bioinformatics/btq064 20179076

[B111] Yu G. Lu C. Wang J. (2017c). NoGOA: predicting noisy GO annotations using evidences and sparse representation. BMC Bioinformatics 18:350. 10.1186/s12859-017-1764-z 28732468 PMC5521088

[B112] Yu G. Luo W. Fu G. Wang J. (2016b). Interspecies gene function prediction using semantic similarity. BMC Syst. Biol. 10:361. 10.1186/s12918-016-0361-5 28155711 PMC5260010

[B113] Yu G. Rangwala H. Domeniconi C. Zhang G. Zhang Z. (2013b). “Protein function prediction by integrating multiple kernels?” in Twenty-Third International Joint Conference on Artificial Intelligence (Beijing), 1869–1875.

[B114] Yu G. Rangwala H. Domeniconi C. Zhang G. Zhang Z. (2015a). Predicting protein function using multiple kernels. IEEE/ACM Trans. Comput. Biol. Bioinformatics 12, 219–233. 10.1109/TCBB.2014.2351821 26357091

[B115] Yu G. Wang K. Domeniconi C. Guo M. Wang J. (2020a). Isoform function prediction based on bi-random walks on a heterogeneous network. Bioinformatics 36, 303–310. 10.1093/bioinformatics/btz535 31250882

[B116] Yu G. Wang K. Fu G. Guo M. Wang J. (2020b). NMFGO: Gene function prediction via nonnegative matrix factorization with gene ontology. IEEE/ACM Trans. Comput. Biol. Bioinformatics 17, 238–249. 10.1109/TCBB.2018.2861379 30059316

[B117] Yu G. Wang K. Fu G. Wang J. Zeng A. (2017d). Protein function prediction based on multiple networks collaborative matrix factorization. J. Comput. Res. Dev. 54, 2660–2673. 10.7544/issn1000-1239.2017.20170644

[B118] Yu G. Wang Y. Wang J. Fu G. Guo M. Domeniconi C. (2018b). “Weighted matrix factorization based data fusion for predicting lncRNA-disease associations?” in IEEE International Conference on Bioinformatics and Biomedicine (Madrid), 572–577. 10.1109/BIBM.2018.8621081

[B119] Yu G. Zhang G. Rangwala H. Domeniconi C. Yu Z. (2012b). “Protein function prediction using weak-label learning?” in Conference on Bioinformatics, Computational Biology and Biomedicine (Orlando, FL), 202–209. 10.1145/2382936.2382962

[B120] Yu G. Zhao Y. Lu C. Wang J. (2017e). HashGO: hashing gene ontology for protein function prediction. Comput. Biol. Chem. 71, 264–273. 10.1016/j.compbiolchem.2017.09.010 29031869

[B121] Yu G. Zhu H. Domeniconi C. (2015b). Predicting protein functions using incomplete hierarchical labels. BMC Bioinformatics 16:1. 10.1186/s12859-014-0430-y 25591917 PMC4384381

[B122] Yu G. Zhu H. Domeniconi C. Guo M. (2015c). Integrating multiple networks for protein function prediction. BMC Syst. Biol. 9:S3. 10.1186/1752-0509-9-S1-S3 25707434 PMC4331678

[B123] Yu G. Zhu H. Domeniconi C. Liu J. (2015d). Predicting protein function via downward random walks on a gene ontology. BMC Bioinformatics 16:271. 10.1186/s12859-015-0713-y 26310806 PMC4551531

[B124] Zeng X. Zhang X. Zou Q. (2015). Integrative approaches for predicting microRNA function and prioritizing disease-related microRNA using biological interaction networks. Brief. Bioinformatics 17, 193–203. 10.1093/bib/bbv033 26059461

[B125] Zhang J. Zhang Z. Chen Z. Deng L. (2019). Integrating multiple heterogeneous networks for novel lncRNA-disease association inference. IEEE/ACM Trans. Comput. Biol. Bioinformatics 16, 396–406. 10.1109/TCBB.2017.2701379 28489543

[B126] Zhang M.-L. Zhou Z.-H. (2014). A review on multi-label learning algorithms. IEEE Trans. Knowl. Data Eng. 26, 1819–1837. 10.1109/TKDE.2013.39

[B127] Zhang X. F. Dai D. Q. Li X. X. (2012). Protein complexes discovery based on protein-protein interaction data via a regularized sparse generative network model. IEEE/ACM Trans. Comput. Biol. Bioinformatics 9, 857–870. 10.1109/TCBB.2012.20 22291160

[B128] Zhang Z. Miller W. Lipman D. J. (1997). Gapped BLAST and PSI-BLAST: a new generation of protein database search programs. Nucleic Acids Res. 25, 3389–3402. 10.1093/nar/25.17.3389 9254694 PMC146917

[B129] Zhao Y. Fu G. Wang J. Guo M. Yu G. (2019a). Gene function prediction based on gene ontology hierarchy preserving hashing. Genomics 111, 334–342. 10.1016/j.ygeno.2018.02.008 29477548

[B130] Zhao Y. Wang J. Guo M. Zhang X. Yu G. (2019b). Cross-species protein function prediction with asynchronous-random walk. IEEE/ACM Trans. Comput. Biol. Bioinformatics 99, 1–12. 10.1109/TCBB.2019.2943342 31562099

[B131] Zhao Y. Wang J. Guo M. Zhang Z. Yu G. (2019c). Protein function prediction based on zero-one matrix factorixation. Sci. Sin. Inform. 49, 1159–1174. 10.1360/N112018-00331

[B132] Zheng Q. Wang X.-J. (2008). GOEAST: a web-based software toolkit for gene ontology enrichment analysis. Nucleic Acids Res. 36, W358?W363. 10.1093/nar/gkn276 18487275 PMC2447756

[B133] Zhou N. Jiang Y. Bergquist T. R. Lee A. J. Kacsoh B. Z. Crocker A. W.. (2019). The CAFA challenge reports improved protein function prediction and new functional annotations for hundreds of genes through experimental screens. Genome Biol. 20, 1–23. 10.1186/s13059-019-1835-8 31744546 PMC6864930

[B134] Zou Q. Sangaiah A. K. Mrozek D. (2019). Machine learning techniques on gene function prediction. Front. Genet. 10:938. 10.3389/978-2-88963-214-5 31636657 PMC6788354

