# Supplementary material for: A Literature Review of Gene Function Prediction by Modeling Gene Ontology
Source: Front Genet. 2020 Apr 24;11:400. doi: 10.3389/fgene.2020.00400 (PMC7193026; doi:10.3389/fgene.2020.00400)
Supplement: Supplementary file 1 [file Data_Sheet_1.pdf]

“A Literature Review of Gene Function Prediction by Modeling Gene Ontology” Supplementary Material

1 DEMO CODES FOR CONSTRUCTING GO DAG

Both the ontology file and the Gene Ontology annotation files of different specices can be accessed from the official website http://geneontology.org/. The ontology file ‘go.obo’ stores the hierarchical relationships between GO terms and provides controlled words to describe the biological meanings. Some exemplars are shown as follows:

```
[Term]
id: GO:0006856
name: eye pigment precursor transport
namespace: biological_process
def: "The directed movement of eye pigment precursors, the inactive
     forms of visual pigments, into, out of or within a cell, or
     between cells, by means of some agent such as a transporter or
     pore." [GOC:ai]
is_a: GO:0006810 ! transport
relationship: part_of GO:0048066 ! developmental pigmentation

[Term]
id: GO:0001531
name: interleukin-21 receptor binding
namespace: molecular_function
def: "Interacting selectively and non-covalently with the interleukin
     -21 receptor." [GOC:ai]
synonym: "IL-21" NARROW []
synonym: "interleukin-21 receptor ligand" NARROW []
is_a: GO:0005126 ! cytokine receptor binding

[Term]
id: GO:0001533
name: cornified envelope
namespace: cellular_component
def: "A type of plasma membrane that has been modified through addition
     of distinct intracellular and extracellular components, including
     ceramide, found in cornifying epithelial cells (corneocytes)."
     [GOC:add, PMID:11112355, PMID:11590230, PMID:15803139]
is_a: GO:0005886 ! plasma membrane
......
```

In this file, each term in namespace (or sub-ontology) cellular_component (CCO), molecular_function (MFO) or biological_process (BPO) is tagged with an ‘id’, ‘name’, ‘origin’ and relationships (i.e., ’is_a’)

with other terms. Based on this ontology file, we can construct a DAG (directed acyclic graph) with moderate scripts, such as ‘geneont’ in Bioinformatics toolbox of Matlab, ‘GO.db’ for R and ‘GOATools’ for Python. In the following two subsections, we give some demo codes using Matlab and R, respectively.

1.1 Using Matlab

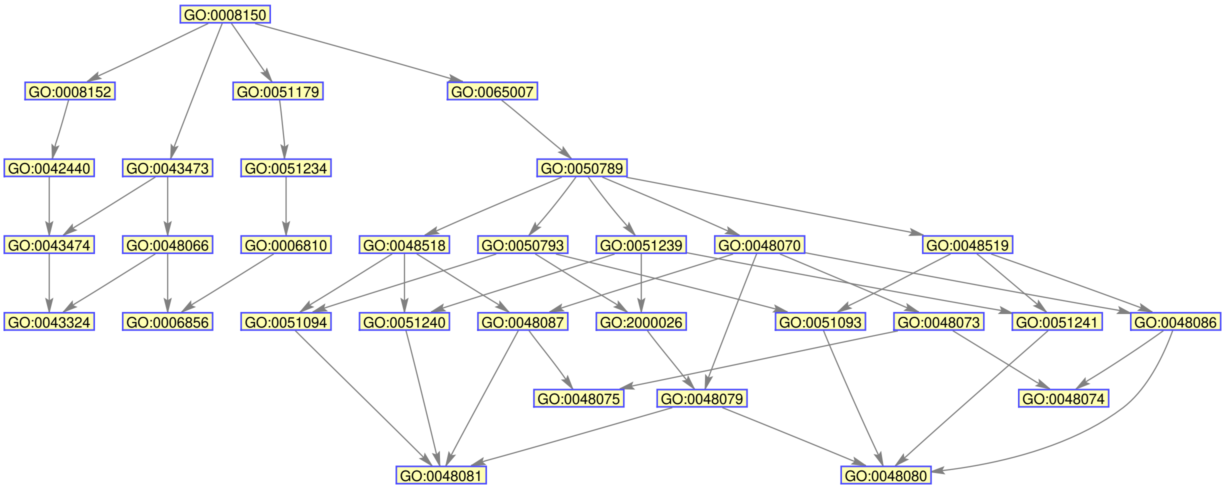

Figure S1. A snapshot of the GO DAG with selected GO terms in Figure 1 of the main text.

By taking the GO terms in BPO in Fig. 1 of the main text as the selected GO terms, with the following Matlab codes, we can plot the selected GO terms and their ancestors in a DAG in Figure S1 with *is a* or *part of* relationships. Other more advanced options for drawing such a DAG can be made by referring to the help documents of Bioinformatics toolbox of Matlab. In addition, ‘getancestors’ and ‘getdescendants’ can obtain a fixed level of ancestor and descendant terms of the selected GO terms, respectively. In this way, a DAG can be plotted with a set of GO terms without direct connections or hierarchical relationships.

```
goObj=geneont(File',go.obo');% Read the ontology file

% Show a snapshot of ontology based on selected terms SelGOs

ancestors=[];
%descendants=[];
for i=1:length(SelGOs)
    goid=SelGOs(i);
    % Retrieve ancestor terms
    ancestors =union(ancestors, getancestors(goObj,goid));
    % Retrieve descendant terms
    %descendants =union(descendants, getdescendants(goObj,goid));
end
% Plot a DAG with ancestor terms of selected GO terms
subontology = goObj(ancestors);
%subontology = goObj(descendants);
rpt = [num2goid(cell2mat(get(subontology.terms,'id')))...
       get(subontology.terms,'name')]';
```

```
disp(sprintf('%s --> %s \n',rpt{:}))
% Get the association matrix of DAG composed of selected GO terms
cm = getmatrix(subontology);

BG = biograph(cm,rpt(1,:));
view(BG); % Show the DAG
```

1.2 Using 'GO.db of R'

Here, we give the demo codes of generating adjacent matrix of GO terms with selected GO terms using 'GO.db'. 'GO.db' is an R package storing a set of term maps, which describe the entire Gene Ontology assembled using data from GO. This package is routinely updated and contains some functions, such as 'GOBPANCESTOR' (obtain biological process ancestors for each GO term in current BPO), GOBPCHILDREN (obtain biological process direct childrens for each GO term in current BPO), GOBPOFFSPRING (obtain biological process descendants for GO term in current BPO) and so on. Here is the demo code.

```
if (!requireNamespace("BiocManager", quietly = TRUE)) # Install GO.db
    install.packages("BiocManager")
BiocManager::install("GO.db")

suppressMessages(library(GO.db)) # Load GO.db

# Get direct childrens for each GO term in current BPO
ALLGO <- as.list(GOBPCHILDREN)

# Read ID of selected GO terms from 'GOID.txt' file
GOID <- read.csv("GOID.txt", sep="")
SelGO<-as.character(GOID$ID)

# Establish the adjacent matrix of the DAG
dagMat<-getDAG(SelGO,ALLGO)
#dagMat1<-getDAG1(SelGO,ALLGO)

# Function
# Equally treat the relationships between GO terms
getDAG<- function(SelGO,ALLGO){
   Nfun <- length(SelGO) # The number of selected terms
   dagMat<-matrix(0,Nfun,Nfun)
   for(i in 1:Nfun){
     dagMat[i,i]<- 1
     goid1<-SelGO[i]
     child<-ALLGO[[goid1]] # Get all direct childrens of goid1
     childGOs<-intersect(child,SelGO)
     if(length(childGOs)>0){
       for(j in 1:length(childGOs)){
         child_idxs<-which(SelGO==childGOs[j])
```

```
        dagMat[i,child_idxs]<- 1
      }
    }
  }
  return(dagMat)
}

#Distinguish the relationships between GO terms
getDAG1<- function(SelGO,ALLGO){
  Nfun <- length(SelGO) # The number of selected terms
  dagMat<-matrix(0,Nfun,Nfun)
  for(i in 1:Nfun){
    dagMat[i,i]<- 1 # 1 represents the term itself
    goid1<-SelGO[i]
    child<-ALLGO[[goid1]] # Get all direct childrens of goid1
    if(length(child)>0){
      for(j in 1:length(child)){
        child_idxs<-which(SelGO==child[j])
        if(length(child_idxs)>0){
          {if(attr(child[j],"names")=="is_a"){
              dagMat[i,child_idxs]<- 2}} # 2 represents "is_a"
          {if(attr(child[j],"names")=="part_of"){
              dagMat[i,child_idxs]<-3}} # 3 represents "part_of"
          {if((attr(child[j],"names")=="negatively_regulates")
          ||(attr(child[j],"names")=="positively_regulates")
          ||(attr(child[j],"names")=="regulates")){
              dagMat[i,child_idxs]<-4}} # 4 represents "regulates"
        }
      }
    }
  }
  return(dagMat)
}
```

By the above demo codes, we can establish an adjacency matrix of GO terms of BPO in Fig. 1 of the main text, and show the matrix in Table S1 (treat the relationships equally) and Table S2 (distinguish the relationships). Based on this association matrix and the direct annotations of genes in the GO annotation file, we can apply the True Path Rule to augment the positive annotations to ancestor terms, and negative ones to descendant terms in gene-term association matrix $\mathbf{Y}$. After that, we can do gene function prediction based on $\mathbf{Y}$.

Table S1. An exemplar adjacency matrix of selected GO terms based on GO DAG, and the relationships (*is a*, *part of* and *regulate*) are equally treated.

		1	2	3	4	5	6	7	8	9	10	11	12	13	14	15	16
1	GO:0008150	1	1	1	0	0	1	1	0	0	0	0	0	0	0	0	0
2	GO:0043473	0	1	0	1	1	0	0	0	0	0	0	0	0	0	0	0
3	GO:0050789	0	0	1	0	0	1	1	0	1	0	0	0	0	0	0	0
4	GO:0043474	0	0	0	1	0	0	0	1	0	0	0	0	0	0	0	0
5	GO:0048066	0	0	0	0	1	0	0	1	1	1	1	1	0	0	0	0
6	GO:0048519	0	0	0	0	0	1	0	0	0	0	1	0	0	0	0	0
7	GO:0048518	0	0	0	0	0	0	1	0	0	0	0	1	0	0	0	0
8	GO:0043324	0	0	0	0	0	0	0	1	0	0	0	0	0	0	0	0
9	GO:0048070	0	0	0	0	0	0	0	0	1	0	1	1	0	0	0	0
10	GO:0006856	0	0	0	0	0	0	0	0	0	1	0	0	0	0	0	0
11	GO:0048086	0	0	0	0	0	0	0	0	0	0	1	0	1	1	0	0
12	GO:0048087	0	0	0	0	0	0	0	0	0	0	0	1	0	0	1	1
13	GO:0048080	0	0	0	0	0	0	0	0	0	0	0	0	1	0	0	0
14	GO:0048074	0	0	0	0	0	0	0	0	0	0	0	0	0	1	0	0
15	GO:0048081	0	0	0	0	0	0	0	0	0	0	0	0	0	0	1	0
16	GO:0048075	0	0	0	0	0	0	0	0	0	0	0	0	0	0	0	1

Table S2. An exemplar adjacency matrix of selected GO terms based on GO DAG, and the relationships (*is a*, *part of* and *regulate*) are denoted as 2, 3 and 4, respectively

		1	2	3	4	5	6	7	8	9	10	11	12	13	14	15	16
1	GO:0008150	1	2	4	0	0	4	4	0	0	0	0	0	0	0	0	0
2	GO:0043473	0	1	0	3	2	0	0	0	0	0	0	0	0	0	0	0
3	GO:0050789	0	0	1	0	0	2	2	0	2	0	0	0	0	0	0	0
4	GO:0043474	0	0	0	1	0	0	0	2	0	0	0	0	0	0	0	0
5	GO:0048066	0	0	0	0	1	0	0	3	4	3	4	4	0	0	0	0
6	GO:0048519	0	0	0	0	0	1	0	0	0	0	2	0	0	0	0	0
7	GO:0048518	0	0	0	0	0	0	1	0	0	0	0	2	0	0	0	0
8	GO:0043324	0	0	0	0	0	0	0	1	0	0	0	0	0	0	0	0
9	GO:0048070	0	0	0	0	0	0	0	0	1	0	2	2	0	0	0	0
10	GO:0006856	0	0	0	0	0	0	0	0	0	1	0	0	0	0	0	0
11	GO:0048086	0	0	0	0	0	0	0	0	0	0	1	0	2	2	0	0
12	GO:0048087	0	0	0	0	0	0	0	0	0	0	0	1	0	0	2	2
13	GO:0048080	0	0	0	0	0	0	0	0	0	0	0	0	1	0	0	0
14	GO:0048074	0	0	0	0	0	0	0	0	0	0	0	0	0	1	0	0
15	GO:0048081	0	0	0	0	0	0	0	0	0	0	0	0	0	0	1	0
16	GO:0048075	0	0	0	0	0	0	0	0	0	0	0	0	0	0	0	1
